# Effects of ACEIs and ARBs on the Residual Renal Function in Peritoneal Dialysis Patients: A Meta-Analysis of Randomized Controlled Trials

**DOI:** 10.1155/2020/6762029

**Published:** 2020-09-23

**Authors:** Lei Ding, Jingjuan Yang, Lizhu Li, Yi Yang

**Affiliations:** Department of Nephrology, The Fourth Affiliated Hospital, College of Medicine, Zhejiang University, N1 Shangcheng Road, Yiwu, Zhejiang, China

## Abstract

**Background:**

In peritoneal dialysis (PD) patients, whether angiotensin-converting enzyme inhibitors (ACEIs) and angiotensin receptor blockers (ARBs) could protect residual renal function is still controversial. To assess the effects of ACEIs and ARBs on the residual renal function and cardiovascular (CV) events in peritoneal dialysis patients, we performed a meta-analysis of randomized controlled trials.

**Materials and Methods:**

We searched PubMed, EMBASE, the Cochrane Library, the CNKI database, and the Wanfang database for relevant articles from database inception to November 30, 2019. Randomized controlled trials were included. The primary outcome was the decline in the residual renal function (RRF).

**Results:**

Thirteen trials with 625 participants were included in the meta-analysis. The average residual GFR declined by 1.79 ml/min per 1.73 m^2^ in the ACEI/ARB group versus 1.44 ml/min per 1.73 m^2^ in the placebo or active control group at 3 mo. The average residual GFR declined by 2.02 versus 2.06, 2.16 versus 2.72, and -0.04 versus 2.74 ml/min per 1.73 m^2^ in the placebo or active control group at 6 months (mo), 12 mo, and 24 mo, respectively. The decline in residual GFR showed a significant difference between the ACEI/ARB group and the placebo or active control group at 12 mo (MD = −0.64 ml/min per 1.73 m^2^; 95% CI: -0.97~-0.32; *I*^2^ = 44%; *P* < 0.0001). No significant difference was observed in Kt/V, urinary protein excretion, weekly creatinine clearance, CV events, or serum potassium levels.

**Conclusions:**

In the present study, we found that the use of ACEIs and ARBs, especially long-term treatment, decreased the decline of RRF in patients on PD. ACEIs and ARBs do not cause an additional risk of side effects.

## 1. Introduction

Cardiovascular (CV) disease is the main leading cause of death in patients with end-stage renal disease (ESRD), which accounts for over 40% deaths in dialysis patients [1]. Previous studies demonstrated that using angiotensin-converting enzyme inhibitors (ACEIs) and angiotensin receptor blockers (ARBs) could reduce the morbidity of cardiovascular events and mortality in chronic kidney disease (CKD) patients [2, 3].

In peritoneal dialysis (PD), the preserved residual renal function (RRF) is significantly associated with the better CV outcomes and lower mortality [4–6], mainly due to better control of malnutrition and hypertension, less ventricular hypertrophy, and lower rates of infection and hospitalization [7, 8]. These date suggest that preserve RRF in PD patients may be critical. Previous studies found that ACEIs and ARBs might preserve the residual renal function via decreasing inflammation and glomerulosclerosis in PD patients [9–11]. However, most studies aimed at evaluating the effects of ACEI and ARB therapy in dialysis patients provided different consequences and much uncertainty about the protective effects of these medications persists [12, 13]. Therefore, there is still no reliable evidence to confirm whether ACEIs/ARBs are worthy of clinical promotion in PD patients.

In the present study, randomized controlled studies (RCTs) using ACEIs/ARBs in PD patients were systematically evaluated in this meta-analysis to evaluate whether ACEIs and ARBs could protect the residual renal function in PD patients and whether they could provide evidence and promote application in PD patients.

## 2. Material and Methods

### 2.1. Date Sources, Search Strategy, and Selection Criteria

We searched PubMed, EMBASE, the Cochrane Library, the CNKI database, and the Wanfang database for relevant articles from database inception to November 30, 2019. We used the MeSH headings and text words of all spellings of known ACEIs and ARBs and peritoneal dialysis (Additional file 1). Randomized controlled trials without language limitations were included.

Eligible studies had the following characteristics: (1) studies of human subjects with RCTs, (2) the subjects in the study were composed of peritoneal dialysis patients over 18 years of age, (3) studies with documented data on renal outcomes or cardiovascular events, and (4) studies with a treatment duration of more than 3 months (mo).

### 2.2. Data Extraction and Quality Assessment

Published reports were obtained for each eligible trial, and relevant information extracted into a spreadsheet. The following data were extracted: first author, study characteristics (publication year, country, duration, setting, and design), and participant characteristics (interventions, age, sex, and sample size). The primary outcome was the decline in RRF. The secondary outcomes were anuria, change of Kt/V, urinary protein extraction, weekly creatine clearance, and cardiovascular events. RRF was measured by the glomerular filtration rate (GFR) or 24-hour urinary urea and creatinine clearances. Anuria was defined as total absence of urine output. Cardiovascular events included death from cardiovascular causes, nonfatal myocardial infarction, cerebrovascular events with permanent neurologic deficit, and peripheral vascular disease requiring lower-limb amputation above the ankle.

The literature were searched and identified by two investigators (LD and JJY) independently. Data extraction and quality assessment were undertaken independently by two investigators (LD and LZL) using a standardized approach. Any disagreement between the two investigators in the abstracted data was adjudicated by a third reviewer (YY). For studies with insufficient information, the reviewers contacted the primary authors, when possible, to acquire and verify the data.

### 2.3. Risk of Bias Assessment

The methodological quality of RCTs was assessed using the Cochrane risk of bias tool.

### 2.4. Statistical Analysis

For each eligible study, dichotomous data were analyzed by using the risk ratio (RR), which was computed using the Mantel-Haenszel method (fixed- or random-effects models). Continuous outcomes measured on the same scale are expressed as a mean value and standard deviation and were analyzed by using weighted mean differences (WMDs). The *I*-squared (*I*^2^) test was performed to assess the impact of study heterogeneity on the results of the meta-analysis. According to the Cochrane review guidelines, if severe heterogeneity was present, indicated by *I*^2^ > 50%, then the random-effect models were chosen. Otherwise, the fixed-effect models were used. A sensitivity analysis was performed if low-quality trials were identified. The overall effect was tested using *Z* scores calculated by Fisher's *Z* transformation, with significance set at *P* < 0.05. Publication bias was assessed with funnel plots. Data analyses were performed by using Review Manager 5.3 (Cochrane Collaboration, Oxford, UK).

## 3. Results

**Figure 1:**
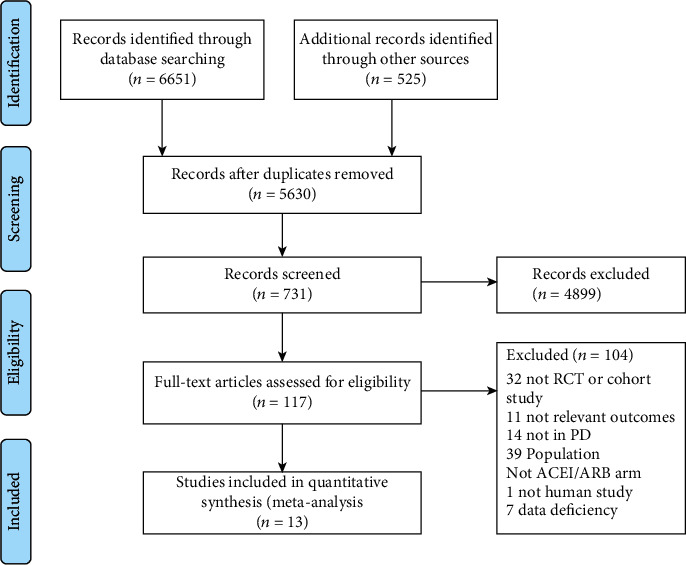
PRISMA 2009 flow diagram.

After searching the electronic databases and selecting the relevant citations, 5630 studies were identified. Browsing the headlines and summaries, we performed full manuscript reviews of the remaining 117 articles. Thirteen reports were included in the meta-analysis (Figure 1).

### 3.1. Baseline Characteristics of the Patients Included

Table 1 describes the characteristics of the 13 included studies with 625 participants in total [9, 10, 14–24]. These studies were performed between 2003 to 2016. Three studies (*n* = 165) compared ACEIs with active controls [9, 18, 23], one study (*n* = 90) compared ACEIs with ARBs [19], eight studies (*n* = 346) compared ARBs with active controls [10, 14–17, 20, 22, 24], and one studies (*n* = 24) compared ARBs with a placebo [21].

### 3.2. Risk of Bias

**Figure 2:**
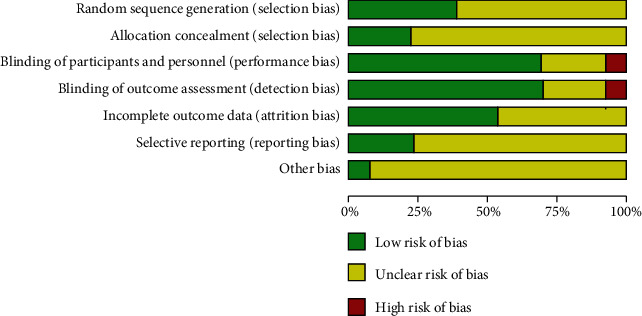
Quality assessment for included trials.

The quality of the included studies was estimated using the Cochrane Collaboration tool for assessing the risk of bias; a low versus high risk of bias is indicated for each study in Figure 2.

### 3.3. Decline of the Residual Renal Function

**Figure 3:**
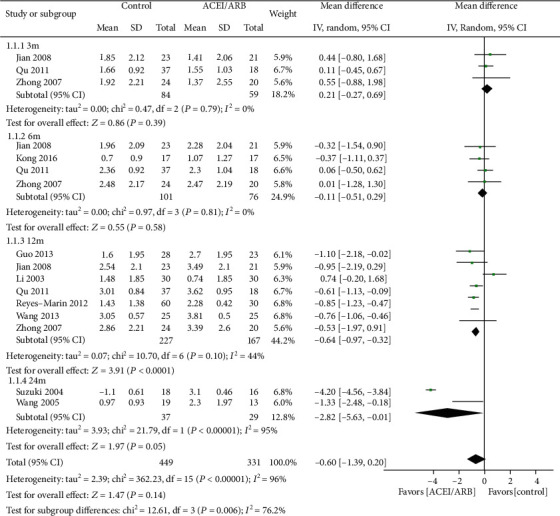
Change of residual GFR in the ACEI/ARB group versus placebo or other active agent group.

Data regarding the effects of ACEIs/ARBs on RRF were available in 10 trials [9, 10, 14, 16–19, 22–24]. The average residual GFR declined by 1.79 ml/min per 1.73 m^2^ in the ACEI/ARB group versus 1.44 ml/min per 1.73 m^2^ in the placebo or active control group at 3 mo. The average residual GFR declined by 2.02 versus 2.06, 2.16 versus 2.72, and -0.04 versus 2.74 ml/min per 1.73 m^2^ in the placebo or active control group at 6 mo, 12 mo, and 24 mo, respectively. The decline in residual GFR showed a significant difference between the ACEI/ARB group and the placebo or active control group at 12 mo (MD = −0.64 ml/min per 1.73 m^2^; 95% CI: -0.97~-0.32; *I*^2^ = 44%; *P* < 0.0001) (Figure 3 and Additional Figure 1).

### 3.4. Anuria

**Figure 4:**
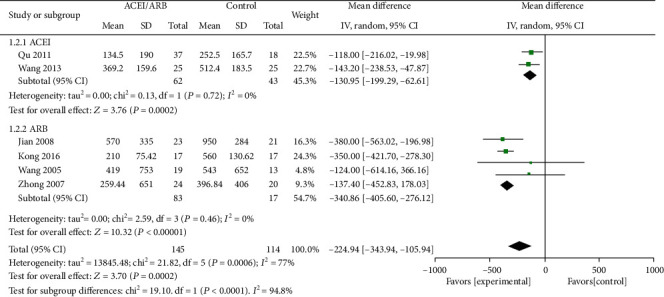
Change of urine volume in the ACEI/ARB group versus placebo or other active agent group.

There were 6 studies described the change in urine volume between the ACEI/ARB group and the control group [16–18, 22–24]. Three studies reported the anuria [9, 22, 24]. The decline in urine volume showed a significant difference between the ACEI/ARB group and the active control group (MD = −224.94 ml/d; 95% CI: -343.94~-105.94; *I*^2^ = 77%; *P* < = 0.0002) (Figure 4). However, there was no significant difference in the effect of anuria (Additional Figure 2).

### 3.5. Change of Kt/V

The average change in Kt/V declined by 0.07 in the ACEI/ARB group versus 0.17 in the placebo or active control group (Additional Figure 3).

### 3.6. Change in Urinary Protein Excretion

The average change in urinary protein excretion declined by -0.10 g/d in the ACEI/ARB group versus 0.65 g/d in the placebo or active control group (Additional Figure 4).

### 3.7. Change of Weekly Creatinine Clearance

The average change in weekly creatinine clearance declined by 2.86 L/wk per 1.73 m^2^ in the ACEI/ARB group versus 7.76 L/wk per 1.73 m^2^ in the placebo or active control group (Additional Figure 5).

## 4. Discussion

In this quantitative systematic review comprising of 13 trials and 625 participants, we found that both ACEIs and ARBs showed a significant benefit in preserving RRF in PD patients at 12 months. There was no significant benefit when short-term ACEIs/ARBs were used (≤6 months). No significant difference was observed in Kt/V, urinary protein excretion, weekly creatinine clearance, or serum potassium levels.

It has been reported that maintenance of RRF is independently correlated with increased survival in ESRD patients [25]. Preservation of RRF is also associated with improved volume and nutritional status, better blood pressure control, reduced erythropoietin requirements, and lower risk of inflammation [26]. Therefore, preservation of RRF might be a potential therapeutic target in patients on PD.

In PD patients, whether ACEIs and ARBs could protect residual renal function is still controversial. In a RCT including 60 incident PD patients by Li et al., the use of ACEI could slow the decline in RRF [9]. Similarly, Suzuki et al. also suggested that patients undergoing PD with ARB preserved RRF, compared with the control groups [10]. In contrast to these beneficial effects of ACEIs or ARBs on the preserved RRF, a recent retrospective observational analysis in the United States demonstrated that ACEI/ARB use might not reduce the decline of RRF in PD patients [27]. Another observational cohort study of patients initiating PD in the Netherlands also found there were no renoprotective effects of ACEIs/ARBs in PD patients [28]. There were many risk factors affecting the RRF in patients on PD, including PD prescription [29], PD solution types [30], volume status [31], use of diuretics [32], and nephrotoxic agents [33]. Many dialysis patients have more than one of these risk factors so that these confounding factors might modify the beneficial effect of ACEIs/ARBs on preservation of RRF in observational studies.

In the present study, we found the long-term use (12 months) of ACEIs/ARBs presented benefit in preserving RRF in PD patients, although there was no significant benefit when short-term ACEIs/ARBs were used (≤6 months). Dialysis patients experience abnormal response of the renin-angiotensin system (RAS), leading to higher incidence of hypertension, which is one of the leading causes of CV disease and mortality [34]. In an analysis of over 110,000 dialysis patients, the benefit of ACEI/ARB usage was greater in patients who use agents for longer duration [35]. Long-term usage of RAS blockade might help to maintain stable blood pressure and inhibit the RAS system, thereby protecting the residual renal function in dialysis patients.

Our systematic review had some strengths. We analyzed the changes in the residual renal function at different time points and compared not only renal function progression but cardiovascular events in PD patients. Compared with previous studies, our review included more RCTs focused on the residual renal function [36, 37]. The present study does have several limitations. First, the number and sample sizes of trials were too small. The observed different effect should be interpreted cautiously. Second, the majority of the included population were Asians, which might limit the generalizability of the findings. In addition, heterogeneity of the outcome measurement and definitions and methodological quality of included studies were likely to profound the effects estimate of the findings. Therefore, high-quality RCTs with large sample size are needed to shed light on the impact of the present findings in PD patients.

## 5. Conclusion

In the present study, we found that use of ACEIs and ARBs, especially long-term treatment, decreased the decline of the residual renal function in patients on PD. ACEIs and ARBs do not cause an additional risk of side effects. Further studies are still needed to shed light on the impact of ACEIs and ARBs on the residual renal function in PD patients.

## Figures and Tables

**Table 1:** Characteristics of studies in meta-analysis.

Trials	Country	Dialysis modality	Treatment	Sample	Age, years	Female, *N* (%)	SBP mmHg	DBP mmHg	Baseline RRF	Kt/V	Urine volume	Follow-up, months	Dialysis duration, months
Guan and Gong 2015 [15]	China	CAPD	Telmisartan	27	51.3 ± 14.2	16 (59.3%)	155 ± 12	88 ± 7		2.1 ± 0.3	926 ± 241	6	7.6 ± 4.0
			Other active agents#	27	49.3 ± 14.4	14 (51.9%)	152 ± 11	85 ± 9		2.0 ± 0.2	932 ± 235	6	8.0 ± 3.8
Guo and He 2013 [14]	China	PD	Valsartan	28	49 ± 18	13 (46.4%)	134 ± 21	85 ± 17	4.7 ± 2.0			12	
			Other active agents#	23	51 ± 17	10 (43.5%)	130 ± 27	92 ± 19	4.8 ± 2.0			12	
Jian et al. 2008 [16]	China	PD	Valsartan	26	45.6 ± 14.7	19 (38%)			4.61 ± 2.41	1.96 ± 0.34	1506 ± 369	12	
			Other active agents#	24	43.4 ± 14.8				4.51 ± 2.34	1.97 ± 0.28	1603 ± 267	12	
Kong et al. 2016 [17]	China	CAPD	Irbesartan	17	64.6 ± 11.7	7 (41.2%)	144.7 ± 37.6	87.5 ± 23.2	4.24 ± 1.02			6	
			Other active agents#	17	65.3 ± 9.5	8 (47.1%)	142.5 ± 35.9	89.4 ± 25.3	4.09 ± 1.46			6	
Li et al. 2003 [9]	HK	CAPD	Ramipril	30	58.0 ± 14.0	11 (36.7%)	151.8 ± 14.5	83.8 ± 10.2	3.55 ± 2.13	2.06 ± 0.63		12	10.7 ± 10.4
			Other active agents#	30	59.1 ± 9.8	11 (36.7%)	150.5 ± 16.7	83.3 ± 11.5	3.74 ± 1.84	2.12 ± 0.53		12	10.3 ± 7.8
Qu et al. 2011 [18]	China	PD	Benazepril	37			159 ± 16.7	89.3 ± 9.3	4.63 ± 0.97	1.89 ± 0.22	763.2 ± 116.0	12	
			Other active agents#	18			155 ± 16.5	91.5 ± 9.5	4.60 ± 1.10	1.89 ± 0.21	751.8 ± 127.3	12	
Reyes-Marin et al. 2012 [19]	USA	APD	Enalapril	30	42.5 ± 18.5	24 (40%)	130 ± 5	80 ± 10	3.65 ± 1.6	1.95 ± 0.1		12	21.6 ± 7.2
			Losartan	30	49.2 ± 19.6	24 (40%)	135 ± 10	70 ± 5	4.1 ± 2.01	2 ± 0.2		12	18 ± 6
			Historical control group	30	48 ± 7.2				3.68 ± 0.48			12	20.6 ± 6
Shigenaga et al. 2009 [20]	Japan	CAPD	Candesartan/valsartan	30	53.0 ± 2.4	11 (36.7%)	159 ± 7	90 ± 6	9.8 ± 0.96			6	40 ± 10
			Other active agents#	15	53.3 ± 3.1		159 ± 7	89 ± 6	9.1 ± 0.9			6	38 ± 12
Suzuki et al. 2003 [21]	Japan	CAPD	Valsartan	14	56 ± 3		155 ± 4	91 ± 2				12	9.4 ± 2.2
			Placebo^∗^	10	57 ± 2		158 ± 2	88 ± 5				12	8.9 ± 3.2
Suzuki et al. 2004 [10]	Japan	CAPD	Valsartan	18	63.5 ± 3.7		165.0 ± 2.5	76.4 ± 3.2	3.2 ± 0.3			24	
			Other active agents#	16	63.5 ± 3.3		166.0 ± 2.6	75.5 ± 3.6	5.9 ± 0.5			24	
Wang and Xiao 2005 [22]	China	CAPD	Vaisartan	19			158 ± 39	101 ± 27	4.85 ± 2.18	2.08 ± 0.66	1050 ± 680	28 ± 13	
			Other active agents#	13			159 ± 41	104 ± 30	4.87 ± 2.55	2.11 ± 0.59	1120 ± 720	28 ± 13	
Wang and Ren 2013 [23]	China	CAPD	Benazepril	25					4.92 ± 0.49		1098.8 ± 178.0	12	
			Other active agents#	25					4.92 ± 0.54		1063.2 ± 210.8	12	
Zhong et al. 2007 [24]	China	PD	Irbesartan	26			133.0 ± 13.2	83.0 ± 12.3	4.54 ± 2.51	1.97 ± 0.27	1304 ± 395	12	
			Other active agents#	20			135.0 ± 15.8	83.0 ± 10.8	4.43 ± 2.87	1.96 ± 0.21	1206 ± 459	12	

Abbreviations: PD: peritoneal dialysis; CAPD: continuous ambulatory peritoneal dialysis; APD: ambulatory peritoneal dialysis. ^a^Expressed as mean ± SD or median (range); ^∗^If systolic BP was above 140 mmHg in the control group, a calcium antagonist, amlodipine (5 mg once daily), was started, and then the dose was increased up to 10 mg; #Antihypertensive agents were allowed except for ACEIs or ARB treatment.

## Supplementary Materials

Supplementary Materials Additional file 1 Pubmed. Additional Figure 1: change of RRF in the ACEI/ARB group versus placebo or other active agent group at mo 12. Additional Figure 2: effect of ACEI or ARB compared with placebo or other active agents on anuria. Additional Figure 3: change of kt/v in the ACEI/ARB group versus placebo or other active agent group. Additional Figure 4: change of urinary protein excretion in the ACEI/ARB group versus placebo or other active agent group. Additional Figure 5: change of weekly creatinine clearance in the ACEI/ARB group versus placebo or other active agent group.
