# Supplementary material for: Effects of ACEIs and ARBs on the Residual Renal Function in Peritoneal Dialysis Patients: A Meta-Analysis of Randomized Controlled Trials
Source: Biomed Res Int. 2020 Sep 23;2020:6762029. doi: 10.1155/2020/6762029 (PMC7532381; doi:10.1155/2020/6762029)

Additional file 1

Pubmed

1. ("peritoneal dialysis"[MeSH Terms] OR ("peritoneal"[All Fields] AND "dialysis"[All Fields]) OR "peritoneal dialysis"[All Fields])
2. ("PD"[All Fields] OR "CAPD"[All Fields] OR "CCPD"[All Fields] OR "APD"[All Fields])
3. ("ACE"[All Fields] OR "ACE1"[All Fields] OR "ACEI"[All Fields] OR "ACEs"[All Fields])
4. ("angiotensin receptor antagonists"[Pharmacological Action] OR "angiotensin receptor antagonists"[MeSH Terms] OR ("angiotensin"[All Fields] AND "receptor"[All Fields] AND "antagonists"[All Fields]) OR "angiotensin receptor antagonists"[All Fields])
5. (("receptors, angiotensin"[MeSH Terms] OR ("receptors"[All Fields] AND "angiotensin"[All Fields]) OR "angiotensin receptors"[All Fields] OR ("angiotensin"[All Fields] AND "receptor"[All Fields]) OR "angiotensin receptor"[All Fields]) AND block[All Fields])
6. ("angiotensin-converting enzyme inhibitors"[Pharmacological Action] OR "angiotensin-converting enzyme inhibitors"[MeSH Terms] OR ("angiotensin-converting"[All Fields] AND "enzyme"[All Fields] AND "inhibitors"[All Fields]) OR "angiotensin-converting enzyme inhibitors"[All Fields] OR ("angiotensin"[All Fields] AND "converting"[All Fields] AND "enzyme"[All Fields] AND "inhibitors"[All Fields]) OR "angiotensin converting

enzyme inhibitors"[All Fields])

7. ("renin-angiotensin system"[MeSH Terms] OR ("renin-angiotensin"[All Fields] AND "system"[All Fields]) OR "renin-angiotensin system"[All Fields] OR ("renin"[All Fields] AND "angiotensin"[All Fields] AND "system"[All Fields]) OR "renin angiotensin system"[All Fields])
8. ("cilazapril"[MeSH Terms] OR "cilazapril"[All Fields])
9. ("enalapril"[MeSH Terms] OR "enalapril"[All Fields])
10. ("lisinopril"[MeSH Terms] OR "lisinopril"[All Fields])
11. ("captopril"[MeSH Terms] OR "captopril"[All Fields])
12. ("fosinopril"[MeSH Terms] OR "fosinopril"[All Fields])
13. ("perindopril"[MeSH Terms] OR "perindopril"[All Fields])
14. ("ramipril"[MeSH Terms] OR "ramipril"[All Fields])
15. ("quinapril"[MeSH Terms] OR "quinapril"[All Fields])
16. ("benazepril"[Supplementary Concept] OR "benazepril"[All Fields])
17. ("trandolapril"[Supplementary Concept] OR "trandolapril"[All Fields])
18. ("spirapril"[Supplementary Concept] OR "spirapril"[All Fields])
19. ("delapril"[Supplementary Concept] OR "delapril"[All Fields])
20. ("moexipril"[Supplementary Concept] OR "moexipril"[All Fields])
21. ("zofenopril"[Supplementary Concept] OR "zofenopril"[All Fields])
22. ("candesartan"[Supplementary Concept] OR "candesartan"[All Fields])
23. ("eprosartan"[Supplementary Concept] OR "eprosartan"[All Fields])
24. ("irbesartan"[MeSH Terms] OR "irbesartan"[All Fields])

25. ("losartan"[MeSH Terms] OR "losartan"[All Fields])

26. ("olmesartan"[Supplementary Concept] OR "olmesartan"[All Fields])

27. ("telmisartan"[MeSH Terms] OR "telmisartan"[All Fields])

28. ("valsartan"[MeSH Terms] OR "valsartan"[All Fields])

29. 1 OR 2

30. 3 OR 4 OR 5 OR 6 OR 7 OR 8 OR 9 OR 10 OR 11 OR 12 OR 13 OR 14 OR 15 OR 16 OR 17 OR 18 OR 19 OR 20 OR 21 OR 22 OR 23 OR 24 OR 25 OR 26 OR 27

31. 28 AND 29

EMBASE

1. (peritoneal AND ('dialysis'/exp OR dialysis))
2. (pd OR capd OR ccpd OR apd)
3. Continuous Ambulatory Peritoneal Dialysis
4. (ACE OR ACE1 OR ACEi OR ACEs)
5. (angiotensin AND receptor AND antagonists)
6. (angiotensin AND converting AND enzyme AND inhibitors)
7. (renin AND angiotensin AND system)
8. (cilazapril)
9. (enalapril)
10. (fosinopril)
11. (lisinopril)
12. (perindopril)

13. (ramipril)

14. (quinapril)

15. (benazepril)

16. (cilazapril)

17. (trandolapril)

18. (spirapril)

19. (delapril)

20. (moexipril)

21. (zofenopril)

22. (candesartan)

23. (eprosartan)

24. (irbesartan)

25. (losartan)

26. (olmesartan)

27. (telmisartan)

28. (valsartan)

29. #1 OR #2 OR #3

30. #4 OR #5 OR #6 OR #7 OR #8 OR #9 OR #10 OR #11 OR #12 OR #13 OR #14 OR #15 OR #16 OR #17 OR #18 OR #19 OR #20 OR #21 OR #22 OR #23 OR #24 OR #25 OR #26 OR #27 OR #28

31. #29 AND #30

Cocharane

1. (peritoneal dialysis)
2. (PD or CAPD or CCPD or APD)
3. (ACE OR ACE1 OR ACEi OR ACEs)
4. (angiotensin receptor antagonists)
5. (angiotensin converting enzyme inhibitors)
6. (renin angiotensin system)
7. (cilazapril)
8. (enalapril)
9. (fosinopril)
10. (lisinopril)
11. (perindopril)
12. (ramipril)
13. (quinapril)
14. (benazepril)
15. (cilazapril)
16. (trandolapril)
17. (spirapril)
18. (delapril)
19. (moexipril)
20. (zofenopril)
21. (candesartan)
22. (eprosartan)

23. (irbesartan)

24. (losartan)

25. (olmesartan)

26. (telmisartan)

27. (valsartan)

28. 1 OR 2

29. 3 OR 4 OR 5 OR 6 OR 7 OR 8 OR 9 OR 10 OR 11 OR 12 OR 13 OR 14 OR 15 OR 16 OR 17 OR 18 OR 19 OR 20 OR 21 OR 22 OR 23 OR 24 OR 25 OR 26 OR 27

30. 28 AND 29

Additional fig 2 Change of RRF in ACEI/ARB group versus placebo or other active agents group at mo 12

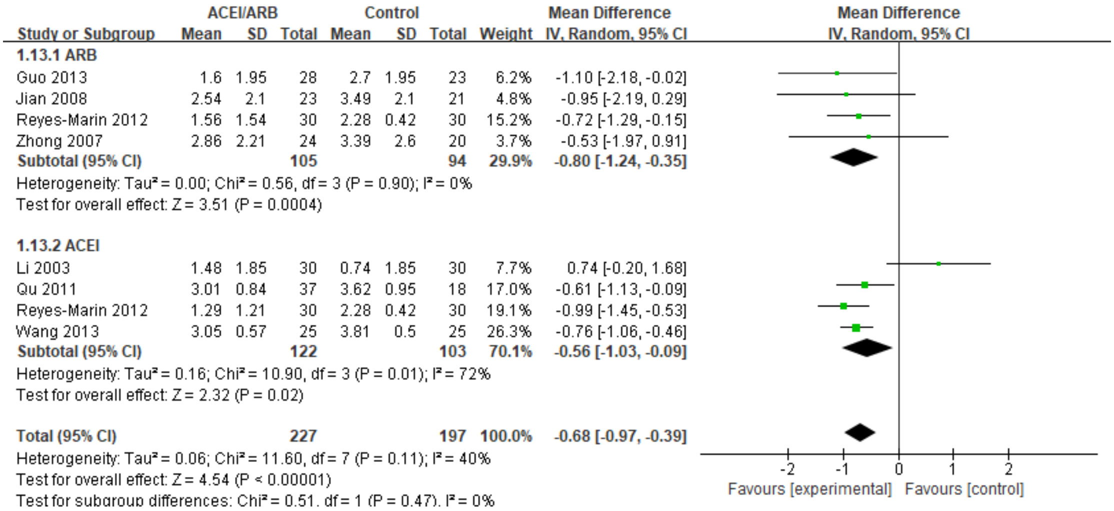

Study or Subgroup	ACEI/ARB Mean	ACEI/ARB SD	ACEI/ARB Total	Control Mean	Control SD	Control Total	Weight	Mean Difference IV, Random, 95% CI
**1.13.1 ARB**								
Guo 2013	1.6	1.95	28	2.7	1.95	23	6.2%	-1.10 [-2.18, -0.02]
Jian 2008	2.54	2.1	23	3.49	2.1	21	4.8%	-0.95 [-2.19, 0.29]
Reyes-Marin 2012	1.56	1.54	30	2.28	0.42	30	15.2%	-0.72 [-1.29, -0.15]
Zhong 2007	2.86	2.21	24	3.39	2.6	20	3.7%	-0.53 [-1.97, 0.91]
**Subtotal (95% CI)**			**105**			**94**	**29.9%**	**-0.80 [-1.24, -0.35]**
Heterogeneity: Tau² = 0.00; Chi² = 0.56, df = 3 (P = 0.90); I² = 0%								
Test for overall effect: Z = 3.51 (P = 0.0004)								
**1.13.2 ACEI**								
Li 2003	1.48	1.85	30	0.74	1.85	30	7.7%	0.74 [-0.20, 1.68]
Qu 2011	3.01	0.84	37	3.62	0.95	18	17.0%	-0.61 [-1.13, -0.09]
Reyes-Marin 2012	1.29	1.21	30	2.28	0.42	30	19.1%	-0.99 [-1.45, -0.53]
Wang 2013	3.05	0.57	25	3.81	0.5	25	26.3%	-0.76 [-1.06, -0.46]
**Subtotal (95% CI)**			**122**			**103**	**70.1%**	**-0.56 [-1.03, -0.09]**
Heterogeneity: Tau² = 0.16; Chi² = 10.90, df = 3 (P = 0.01); I² = 72%								
Test for overall effect: Z = 2.32 (P = 0.02)								
**Total (95% CI)**			**227**			**197**	**100.0%**	**-0.68 [-0.97, -0.39]**
Heterogeneity: Tau² = 0.06; Chi² = 11.60, df = 7 (P = 0.11); I² = 40%								
Test for overall effect: Z = 4.54 (P < 0.00001)								
Test for subgroup differences: Chi² = 0.51, df = 1 (P = 0.47), I² = 0%								

Additional fig 3 Change of urinary protein excretion in ACEI/ARB group versus placebo or other active agents group

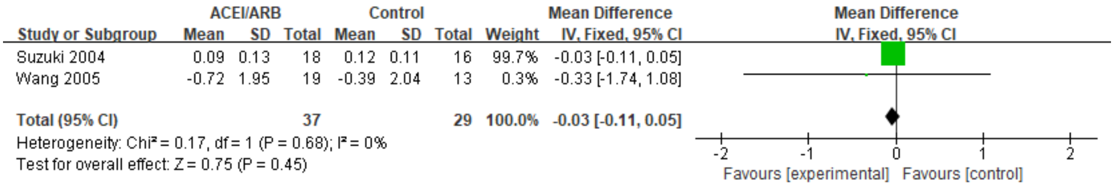

Study or Subgroup	ACEI/ARB Mean	ACEI/ARB SD	ACEI/ARB Total	Control Mean	Control SD	Control Total	Weight	Mean Difference IV, Fixed, 95% CI
Suzuki 2004	0.09	0.13	18	0.12	0.11	16	99.7%	-0.03 [-0.11, 0.05]
Wang 2005	-0.72	1.95	19	-0.39	2.04	13	0.3%	-0.33 [-1.74, 1.08]
**Total (95% CI)**			**37**			**29**	**100.0%**	**-0.03 [-0.11, 0.05]**
Heterogeneity: Chi² = 0.17, df = 1 (P = 0.68); I² = 0%								
Test for overall effect: Z = 0.75 (P = 0.45)								

Additional fig 4 Change of weekly creatinine clearance in ACEI/ARB group versus placebo or other active agents group

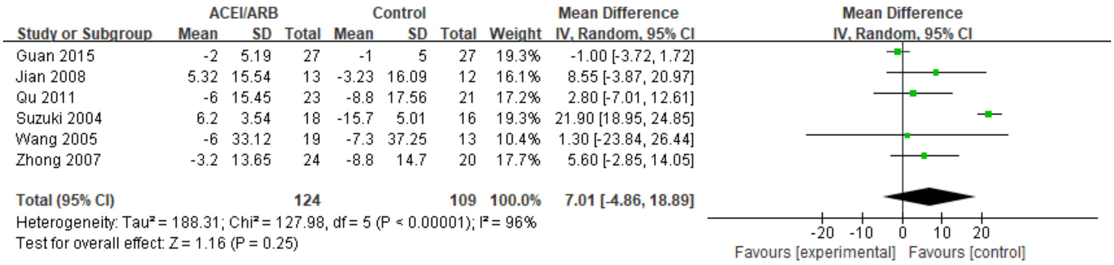

Study or Subgroup	ACEI/ARB Mean	ACEI/ARB SD	ACEI/ARB Total	Control Mean	Control SD	Control Total	Weight	Mean Difference IV, Random, 95% CI
Guan 2015	-2	5.19	27	-1	5	27	19.3%	-1.00 [-3.72, 1.72]
Jian 2008	5.32	15.54	13	-3.23	16.09	12	16.1%	8.55 [-3.87, 20.97]
Qu 2011	-6	15.45	23	-8.8	17.56	21	17.2%	2.80 [-7.01, 12.61]
Suzuki 2004	6.2	3.54	18	-15.7	5.01	16	19.3%	21.90 [18.95, 24.85]
Wang 2005	-6	33.12	19	-7.3	37.25	13	10.4%	1.30 [-23.84, 26.44]
Zhong 2007	-3.2	13.65	24	-8.8	14.7	20	17.7%	5.60 [-2.85, 14.05]
**Total (95% CI)**			**124**			**109**	**100.0%**	**7.01 [-4.86, 18.89]**
Heterogeneity: Tau² = 188.31; Chi² = 127.98, df = 5 (P < 0.00001); I² = 96%								
Test for overall effect: Z = 1.16 (P = 0.25)								

Additional fig 5 Change of EF% in ACEI/ARB group versus placebo or other active agents group

Study or Subgroup	ACEI/ARB Mean	ACEI/ARB SD	ACEI/ARB Total	Control Mean	Control SD	Control Total	Weight	Mean Difference IV, Random, 95% CI
Shigenaga 2009	6	6.24	30	2	7.21	15	45.3%	4.00 [-0.28, 8.28]
Suzuki 2003	5	4.36	14	5	4.36	10	54.7%	0.00 [-3.54, 3.54]
**Total (95% CI)**			**44**			**25**	**100.0%**	**1.81 [-2.09, 5.71]**

Heterogeneity: Tau² = 3.99; Chi² = 1.99, df = 1 (P = 0.16); I² = 50%
Test for overall effect: Z = 0.91 (P = 0.36)

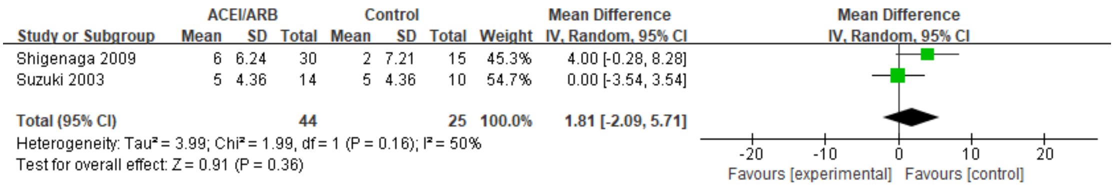

Additional fig 6 Change of serum potassium in ACEI/ARB group versus placebo or other active agents group

Study or Subgroup	ACEI/ARB Mean	ACEI/ARB SD	ACEI/ARB Total	Control Mean	Control SD	Control Total	Weight	Mean Difference IV, Fixed, 95% CI
Jian 2008	-0.13	0.61	23	0.04	0.72	21	18.5%	-0.17 [-0.57, 0.23]
Qu 2011	0.04	0.57	37	-0.06	0.53	18	31.1%	0.10 [-0.21, 0.41]
Zhong 2007	0.24	0.48	24	0.09	0.33	20	50.4%	0.15 [-0.09, 0.39]
**Total (95% CI)**			**84**			**59**	**100.0%**	**0.08 [-0.10, 0.25]**

Heterogeneity: Chi² = 1.87, df = 2 (P = 0.39); I² = 0%
Test for overall effect: Z = 0.86 (P = 0.39)

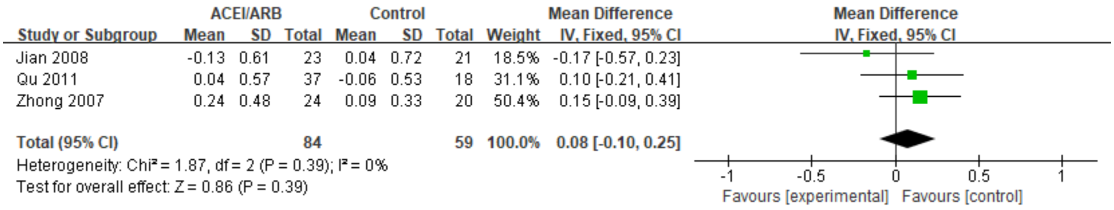

Supplement: Supplementary Materials — Additional file 1 Pubmed. Additional Figure 1: change of RRF in the ACEI/ARB group versus placebo or other active agent group at mo 12. Additional Figure 2: effect of ACEI or ARB compared with placebo or other active agents on anuria. Additional Figure 3: change of kt/v in the ACEI/ARB group versus placebo or other active agent group. Additional Figure 4: change of urinary protein excretion in the ACEI/ARB group versus placebo or other active agent group. Additional Figure 5: change of weekly creatinine clearance in the ACEI/ARB group versus placebo or other active agent group. [file 6762029.f1.pdf]
